# Micronization-assisted pulsed electric field treatment on functional and structural modification of red pepper seed protein isolates

**DOI:** 10.3389/fnut.2026.1936132

**Published:** 2026-10-13

**Authors:** Murtaza Ali, Muhammad Faisal Manzoor, Xin-An Zeng, Muhammad Talha Afraz, Isam A. Mohamed Ahmed

**Affiliations:** 1 Guangdong Provincial Key Laboratory of Intelligent Food Manufacturing, School of Food Science and Engineering, Foshan University, Foshan, China; 2 School of Food Science and Engineering, South China University of Technology, Guangzhou, China; 3 Department of Food Science & Nutrition, College of Food and Agricultural Sciences, King Saud University, Riyadh, Saudi Arabia

**Keywords:** functional properties, micronization, protein isolate, pulsed electric field, red pepper seed, structural properties

## Abstract

**Background:**

Red pepper seeds are an underutilized source of plant proteins with functional potential, but their poor solubility and limited functionality restrict food applications. This study investigated the effects of micronization-assisted pulsed electric field (PEF) treatment on the structural, physicochemical, and functional properties of red pepper seed protein isolates (RPSPIs).

**Methods:**

RPSPIs were subjected to micronization pretreatment followed by PEF treatment under different electric field strengths and processing conditions. The effects of combined treatment on surface hydrophobicity, turbidity, Free Sulfhydryl (SH) Content, particle size distribution, surface morphology, secondary conformation, thermal stability, solubility, emulsifying properties, and foaming characteristics were systematically evaluated. Structural modifications were characterized using Fourier-transform infrared spectroscopy (FTIR), fluorescence spectroscopy, scanning electron microscopy (SEM), X-ray diffraction (XRD) analysis and differential scanning calorimetry (DSC).

**Results:**

The combined treatment significantly impacted the functional properties, including zeta potential and particle size of RPSPI-treated samples, enhancing stability compared to control samples. The structural data revealed important molecular modifications, including enhanced SH^−^ content and reduced fluorescence intensity, suggesting that the structure partially unfolded. In addition, FTIR analysis combined with Amide I deconvolution demonstrated that MR and PEF treatments significantly altered the secondary structure composition of RPSPI, with changes in α-helix, β-sheet, β-turn, and random coil components. The combined MR-PEF treatment promoted greater protein conformational rearrangement, which was associated with improved dispersion behavior, and enhanced functional properties. SEM micrographs revealed significant changes and fragmentation, further supporting our findings. The XRD analysis revealed changes in crystalline structure and crystallinity, while DSC results demonstrated increased denaturation temperatures after treatment, indicating enhanced thermal stability of RPSPI. The combined treatments also had significant impacts on these structural changes, enhancing foaming capacity, solubility, and emulsifying properties and decreasing turbidity.

**Conclusion:**

This study's findings add value to red pepper seed by-product and provide insight into novel processing methods for high-value plant protein components.

## 1 Introduction

In recent years, the growing global demand for alternative and sustainable protein sources has driven the use of potential byproducts as functional food ingredients (1). Among agricultural products, vegetable and fruit seeds are mostly discarded during processing, even though they contain high levels of biomolecules, specifically proteins, techno-functional properties, and lipids. In addition, pepper seeds, a byproduct of Capsicum, are an essential source of plant proteins and offer significant potential functional, nutritional, and health benefits. Previously, research studies have shown that pepper seeds comprise roughly 16%−21% protein and contain a significant amount of essential amino acids, suggesting their potential as an emerging byproduct for food applications as a plant-derived protein source (2).

Recently, red pepper seed protein isolates (RPSPI) derived from pepper seeds have attracted significant interest among researchers due to their functional properties, including foaming and emulsifying properties. On the other hand, native RPSPI exhibits limited solubility and structural resistance like other plant-based proteins, which restricts its functional efficacy in food applications. Additionally, structural properties such as a compact internal structure and strong interactions among internal molecules restrict its exposure to hydrophobic or hydrophilic groups, which can adversely affect its functionality (3). Therefore, numerous conventional approaches, such as thermal denaturation, enzymatic treatment, and high-pressure processing, have been used to enhance the techno-functional properties of RPSPI. Recently, research studies have revealed that physical processing techniques can significantly alter the RPSPI's secondary and tertiary structures while enhancing its techno-functional characteristics, thereby expanding its potential in food applications (4).

Most recently, among emerging food processing methods, non-thermal methods (pulsed electric field (PEF), ultrasound, cold plasma, and micronization) have emerged as protein structural modification methods, demonstrating their ability while showing minimal thermal reduction (5–7). Among these non-thermal processing methods, pulsed electric field (PEF) treatment has been most widely explored for its potential to modify natural macromolecules (8). In addition, PEF processing methods comprise the application of high or low-voltage pulses that generate electric fields capable of modifying molecular interactions within proteins. In addition, exposure of proteins to strong electric fields can disrupt electrostatic interactions, hydrophobic interactions, and hydrogen bonds, causing proteins to unfold and expose to reactive functional groups (9). These structural modifications may alter the techno-functional and physicochemical properties of proteins, such as emulsification, gelation, and solubility (10).

Furthermore, in recent years, particle size reduction methods have also been explored to alter the functionality of plant-based proteins. Among particle size reduction process, micronization is one of the most effective processes for changing functional properties of protein. MR can be achieved using various size reduction processes, including ball milling, jet milling, high pressure homogenization and ultrasonication. These approaches reduce the protein particle size macro to micrometers, enhancing their surface area and improving the exposure and accessibility of reactive groups and active surface. These structural changes might enhance protein-water interactions, influencing functional properties such as foaming capacity solubility and emulsifying activity. Further, reducing particle size may also enhance the functional properties of proteins, such as improving their interactions with surrounding solvents or with the food matrix (11). Nevertheless, MR initially alters the protein's physicochemical properties and might not have much effect on molecular-level modifications (12).

Recent advances in food processing research have revealed that integrating a physical particle size reduction method with emerging non-thermal processing is a promising approach for impactful protein techno-functional modification. In addition, combining MR processing may enhance the accessibility of protein structure, ultimately improving the efficacy of subsequent treatments like PEF (13). Integrating PEF and MR processing methods could change the physical and chemical structures of plant protein isolates, making them more useful in food applications (14). Although there is greater interest in emerging methods for protein modification, few studies have evaluated the effect of MR and PEF on pepper seed protein isolates. Additionally, investigating such integrated approaches could contribute to the development of sustainable, efficient methods for generating functional protein ingredients that are more readily implemented in food formulation.

Therefore, the objective of the current study was to evaluate the combined impact of MR and PEF processing methods on RPSPI. Previously, studies have shown the effects of conventional or single treatment on protein modification. The novelty of the study is that this work is among the first to evaluate the combined effect of MR and PEF treatments on the structural and functional properties of RPSPI. Specifically, emphasis is placed on exploring the effects of these treatments on protein structural alterations and on understanding their potential to enhance the functional properties of RPSPI in food systems. In addition, this study contributes to the current knowledge by elucidating the relationship between MR and PEF-induced protein unfolding in an underutilized plant protein source. Therefore, this study not only broadens the scope of PEF-assisted modification of plant proteins but also establishes a scientific foundation for converting RPS byproducts into value-added functional constituents. Furthermore, this study contributes to the value of pepper seed byproducts and provides insight into novel processing methods for high-value plant protein components.

## 2 Materials and methods

### 2.1 Materials

N-hexane, NaOH, and HCl were acquired from Tianjin Zhi Yuan Reagent Co., Ltd. Tianjin, China, and red pepper seeds were purchased from Jiangmen, China. Sodium dodecyl sulfate, BSA, DPPH assay, and ethanol were purchased from Aladdin Reagent Co., Ltd., Shanghai, China. Additionally, all the other chemicals used in the experiments were of analytical grade and used as supplied without further purification. In addition, the water used in this work was purified with a Milli-Q water purification system (Millipore, Brussels, Belgium), and all glassware was cleaned with distilled water and dried before use.

### 2.2 Extraction and defatting of red pepper seed protein isolates (RPSPI)

Red pepper seeds (RPS) were ground with a high-speed electric grinder, and the ground powder was sieved through a 40 mm sieve. Further, the red seed powders were defatted with n-hexane at a 1:5 (w/v) ratio for 24 h of continuous stirring. Then the defatted powder was desiccated at room temperature, followed by further drying at 50 °C for 2 h in an oven. After that, the dried powder was packed in airtight polythene zip bags and stored at room temperature for further analysis. Furthermore, RPS protein was extracted using an alkali-acid precipitation procedure, with slight modification, as described by Deng et al. (15). Briefly, 200 g of defatted RPS powder was dispersed in 4 L of distilled water (1:20), and the suspension was adjusted to pH 12. The mixture was then subjected to alkaline extraction, followed by acid precipitation to obtain the RPSPI. Subsequently, the alkaline mixture was stirred at ambient temperature for 6 h, then centrifuged at 9,000 × g for 10 min at 4 °C. After filtration, the supernatant was centrifuged for 5 min at 4 °C, and the pH was adjusted to 4.5. After washing the precipitate with distilled water, the pH was adjusted to 7, and then the precipitate was dissolved in 100 mL of distilled water. In addition, the precipitates were dialyzed against ultrapure water using a 3,500 Da molecular weight cutoff membrane for 24 h, then the RPSPI solution was freeze-dried at −80 °C for 12 h, lyophilized, and stored at −20 °C until further analysis. The RPSPI extraction yield based on defatted flour DW was 5.98 ± 0.08% (w/w,) with a protein purity of 80.11 ± 0.12%.

### 2.3 Micronization (MR) and pulsed electric field (PEF) treatment

The RPSPI powders were dissolved in distilled water (5 mg/mL) and well homogenized. The mixture was subjected to the high-speed mechanical shearing MR process using a T 25 Ultra Turrax^®^ (Guangzhou Shenhua Biotechnology, China) at 14,000 rpm for 3 min. On the other hand, a continuous PEF processing system (SY-Z-500, Guangzhou Pahu Technology Co., China; input voltage 220 V/50 Hz, output voltage 0–20 kV, power 2.5 kW) was used in this study. The system delivered bipolar quasi-square wave pulses with an effective pulse width of 43 μs and a frequency of approximately 1.026 kHz, as confirmed by oscilloscope monitoring (Micsig, China; model STO1104C) with a 100 × high-voltage differential probe connected directly across the treatment chamber electrodes. The oscilloscope was set to a 100 V/div vertical scale, a 500 μs/div horizontal scale, and single-sequence acquisition mode. The effective pulse width was measured at half-height of the voltage signal, excluding rise and fall times. The treatment chamber consisted of two plate electrodes with a 0.30 cm gap, an effective electrode area of 13.8474 cm^2^, and a chamber volume of 0.02 mL. A peristaltic pump delivered 150 mL of aqueous protein solution into the PEF system at a flow rate of 200 mL/min after the sample had been brought to room temperature. The temperature of the treated samples was maintained at 20–30 °C during treatment using a water-circulating condensation system set to 4 °C. In short, the samples were treated with a continuous PEF system for 2 min at 15 kV/cm and 20 kV/cm. The specific energy inputs were 19.21 J/mL at an electric field strength of 15 kV/cm and 35.15 J/mL at 20 kV/cm, respectively. For the combined MR and PEF, the samples were treated with MR and then subjected to PEF. The treated samples were denoted as the untreated sample (C), micronization (MR), 15 kV/cm (PEF15), 20 kV/cm (PEF20), MR+15 kV/cm (MR-PEF15), and MR+20 kV/cm (MR-PEF20). Finally, the treated samples were lyophilized and stored in a desiccator at room temperature for further assessment.

### 2.4 SEM

The ion sputtering method was applied to observe the structural modification of treated RPSPI samples using SEM (Thermo Fisher Scientific, Quattro S, Brno s.r.o., Vlastimila Pecha, Czech Republic). Briefly, RPSPI samples were fixed with double-sided sticky tape and assigned to SEM stubs with approximately 8 nm of gold coating. The Carsington sputter coater (Model 108 auto) was used to sputter gold onto the samples for 210 s with 20 mA. The samples were examined with an acceleration voltage of 10 kV at 1,000 ×, and a working distance (WD) of 9.9–12.0 mm (15).

### 2.5 FTIR

The FTIR spectra of treated RPSPI were examined with an FTIR spectrometer (Spectrum 3, PerkinElmer, USA). In short, the treated samples at 1:100 (w/w) were blended with the KBR mixture and pressed into pellets. After that, the FTIR spectra were examined over 4,000–400 cm^−1^ with 64 scans at 4 cm^−1^ resolution (15).

### 2.6 DSC

The thermal characteristics were examined using DSC (DSC-8000, PerkinElmer, Shelton, CT, USA). In short, 2 mg of the samples were well mixed with 6 μL deionized water and packed hermetically in an aluminum pan. After that, the samples were heated and equilibrated at 10 °C/min, ranging from 30 to 150 °C, and an empty alumimium pan was used as a reference, while the denaturation temperature (*T*_d_), enthalpy changes (ΔH), and onset temperature (*T*_0_) were observed using DSC software (TA Instruments, New Castle, DE, USA) (3).

### 2.7 XRD

The treated RPSPI sample's X-ray diffraction (XRD) spectral data were determined using a Bruker D8 DISCOVER diffractometer with Cu Kα radiation (λ = 1.5406 Å). In addition, the data were collected in the 2θ range of 3–90° with a step size of 0.05° and a counting time of 0.5 s per step.

### 2.8 Fluorescence spectroscopy

The intrinsic fluorescence spectra of the RPSPI were measured using a fluorescence spectrophotometer (Hitachi F-4600, Japan). Briefly, the samples were adjusted to a protein concentration of 3 mg/mL and homogenized before analysis. The fluorescence emission spectra were recorded over the wavelength range of 290–500 nm using an excitation wavelength of 280 nm and a slit width of 2.5 nm (15).

### 2.9 Particle size and zeta-potential

Particle size characterization was performed by dynamic light scattering (DLS) using a Zetasizer Ultra (Malvern Panalytical, UK). Samples were dispersed in deionized water and equilibrated before measurement. The Z-average hydrodynamic diameter and polydispersity index (PDI) were calculated using cumulants analysis. The particle-size distribution (PSD) profiles were additionally obtained from the same DLS measurements and evaluated on a volume basis using the instrument software. For zeta potential, 5 mL of the samples were centrifuged at 7,000 × g for 5 min. After centrifugation, 1 mL of the mixture was analyzed with a DTS1070 potential sample cell for Zeta potential (Zetasizer Ultra, Malvern Panalytical) at 25 °C, using water as a blank (15).

### 2.10 Solubility

The treated mixture's solubility was determined by taking a 5 mg/mL sample after homogenizing for 15 min at 8,000 × g. The centrifuged mixture's supernatant was collected, and the BSA procedure was used to determine solubility. In addition, the protein concentration in the supernatant, as compared to the original was calculated using the Equation 1 (16)


$$\begin{array}{l} Protein\;solubility\;=\frac{Csup}{Ctol\;\times 90.8\%}\times 100 \end{array}$$ (1)

where *Csup* denotes the concentration of protein in the supernatant, while *C*_tol_ represents the protein concentration in the solution (1 mg/mL). The Kjeldahl method was used to calculate the protein content at 90.80.

### 2.11 Turbidity

The turbidity of the treated RPSPI samples was evaluated using the prescribed method by Manzoor et al. (4) with slight modifications. Briefly, the RPSPI dispersions absorbance was measured at 400 nm using a UV-spectrophotometer, with deionized water as the blank. The absorbance at 400 nm was used as an indirect measure of turbidity because suspended protein particles scatter incident light. Thus, the absorbance at 400 nm was used as an indicator of turbidity, with higher absorbance corresponding to higher turbidity.

### 2.12 Foam stability (FS) and capacity (FC)

For the determination of FS and FC, 1% (w/v) of the treated samples was added to distilled water at neutral pH for 1 h. After mixing well, 15 mL of the mixture was homogenized for 3 min, and the total volume (mL) was calculated with a graduated cylinder. Then, to calculate the FS and FC, the starting foam layer volumes at 15 and 30 min were added, the following Equations 2 and 3 were used to calculate the FC and FS values (4)


$$\begin{array}{l} FC\;\%=\frac{Foam\;volume}{15\;mL\;}\;\times 100 \end{array}$$ (2)

$$\begin{array}{l} FS\;\%=\frac{Foam\;volume\;after\;15\;and\;30\;\min}{\;mL\;Initial\;foam\;volume\;}\;\times 100. \end{array}$$ (3)

### 2.13 Emulsion activity index (EAI) and stability index (ESI)

The EAI and ESI values of the treated RPSPI were evaluated by adding 3 mL of a RSPSI (0.1%, w/v) mixture to 3 mL of pepper seed oil. After that, the mixture was well homogenized for 2 h at 200 rpm, and from the bottom of the mixture, 5 μL of the emulsion was separated. Then, it was vortexed by adding 5 mL of 0.1% sodium dodecyl sulfate. Further, to assess EAI against a 0.1% SDS solution as a blank, the absorbance of the RPSPI (A_o_) was observed at 500 nm. On the other hand, to evaluate the ESI, the sample absorbance (A_10_) was measured by repeating the scan every 10 min. The EAI and ESI values were calculated using the following Equations 4 and 5 (4)


$$\begin{array}{l} EAI\;\frac{m2}{g}=\frac{2\;\times \;2.303\;\times D\;\times \;Ao}{\;C\;\times \;\emptyset \;\times \;10000\;} \end{array}$$ (4)

$$\begin{array}{l} ESI\;\%=\frac{A10}{\;Ao\;}\;\times 100 \end{array}$$ (5)

where *A*0 is the absorbance at 500 nm, D represents the dilution factor, C is the protein concentration (g/mL), and ϕ is the volume fraction of oil in the emulsion.

### 2.14 Surface hydrophobicity (*H*_0_) analysis

The *H*_0_ values of the RPSPI-GA samples were evaluated using the 1-anilinonaphthalene-8-sulfonic acid (ANS) fluorescent method 0.1 to 0.5 mg/mL of the samples were diluted in distilled water, and then 8 mM of 20 μL ANS was added. The fluorescence intensity of the mixture was measured at an excitation wavelength of 465 nm, an emission wavelength of 360 nm, and excitation and emission slit widths of 5 nm. The slopes of the curve corresponded to the *H*_0_ index of the protein.

### 2.15 Free sulfhydryl (SH) content

The SH content of the sample was evaluated using the prescribed method by Manzoor et al. (4). Briefly, a buffer solution was prepared (0.09 M Gly, 0.086 M Tris, 4 mM EDTA, and pH 7). The sample was mixed with a buffer solution at a dilution of 5 mg/mL for 1 h, then centrifuged at 10,000 × for 15 min. After that, 3 mL of the supernatant was mixed with 30 μL of DTNB and incubated for 15 min. Finally, the absorbance was measured at 412 nm using a UV spectrophotometer (EU-2600 Amodel Onlab, Shanghai, China), and the SH content was calculated using a molar extinction coefficient of 13,600 M^−1^. The results were presented as μmol/g protein, with the buffer solution used as a blank.

### 2.16 Statistical analysis

RPSPI was prepared from a single extraction batch. Each analytical measurement was performed in three technical replicates, and the results are expressed as mean ± standard deviation (SD) (*n* = 3). The findings were statistically examined using one-way ANOVA, and data visualization was performed with OriginPro 8 (Stat-Ease Inc., Minneapolis, USA). The Statistical analysis was conducted using one-way analysis of variance, and significant differences (*p* < 0.05) among the mean values were assessed using Tukey's HSD test for comparisons between the treatment and control samples.

## 3 Results and discussions

### 3.1 Particle size distribution (PSD)

Figure 1 represents the impact of micronization (MR), pulsed electric field (PEF), and their combined treatment on the PSD of red pepper seed protein isolate (RPSPI). The PSD curves, which represent the contribution of various hydrated particle populations following dispersion, were derived from a volume- based distribution analysis. The outcomes revealed that MR, PEF, and their combined treatment significantly (*p* < 0.05) modified the PSD of the RPSPI. The outcomes revealed that the control sample results showed a sharp and concentrated peak mainly distributed within approximately 300–1,100 nm, indicating the presence of relatively compact native protein particles. In contrast, the sample treated with MR and PEF presented broader PSD profiles with reduced peak intensity and increased contribution from larger hydrodynamic populations. On the other hand, the treated samples showed the PSD curves shifted toward larger particles and weaker peaks. These modifications indicate redistribution of particle populations and modification of aggregation behavior rather than a simple increase in overall particle size. These types of modifications are commonly observed in plant proteins, where physical intervention enhances intermolecular interactions in aqueous conditions by altering the native structure (17, 18). The visible shift in PSD peaks toward bigger size regions should be taken together with DLS-derived particle size values listed in Table 1. The average hydrodynamic diameter reduced significantly from 2,144 nm for the control sample to 1,771 nm (MR), 1,523 nm (PEF15), 1,376 nm (PEF20), 521.7 nm (MR-PEF15), and 547.7 nm (MR-PEF20). These findings indicate that MR and PEF treatments efficiently disrupted large native protein aggregates and lowered the average hydrodynamic diameter. However, the PSD profiles indicate the presence of various particle populations, including smaller dispersed particles and a fraction of larger hydrated aggregates formed during structural rearrangement. Further, the difference between the PSD profile and the average DLD particle size values can be explained by the various evidence provided by these measurements. The DLS-derived particle size is the average hydrodynamic diameter of particles dispersed in aqueous solution, whereas the volume-based PSD profile describes the relative distribution of particle population of various hydrodynamic sizes. Since larger particles influence enormously volume-based analysis, even a relatively small fraction of larger hydrated aggregates can produce a visible influence in the larger-size region. Consequently, the presence of larger populations in the PSD curves does not suggest an increase in average particle size, instead, it indicates changes in particle distribution and aggregation state after MR and PEF treatment.

**Figure 1:**
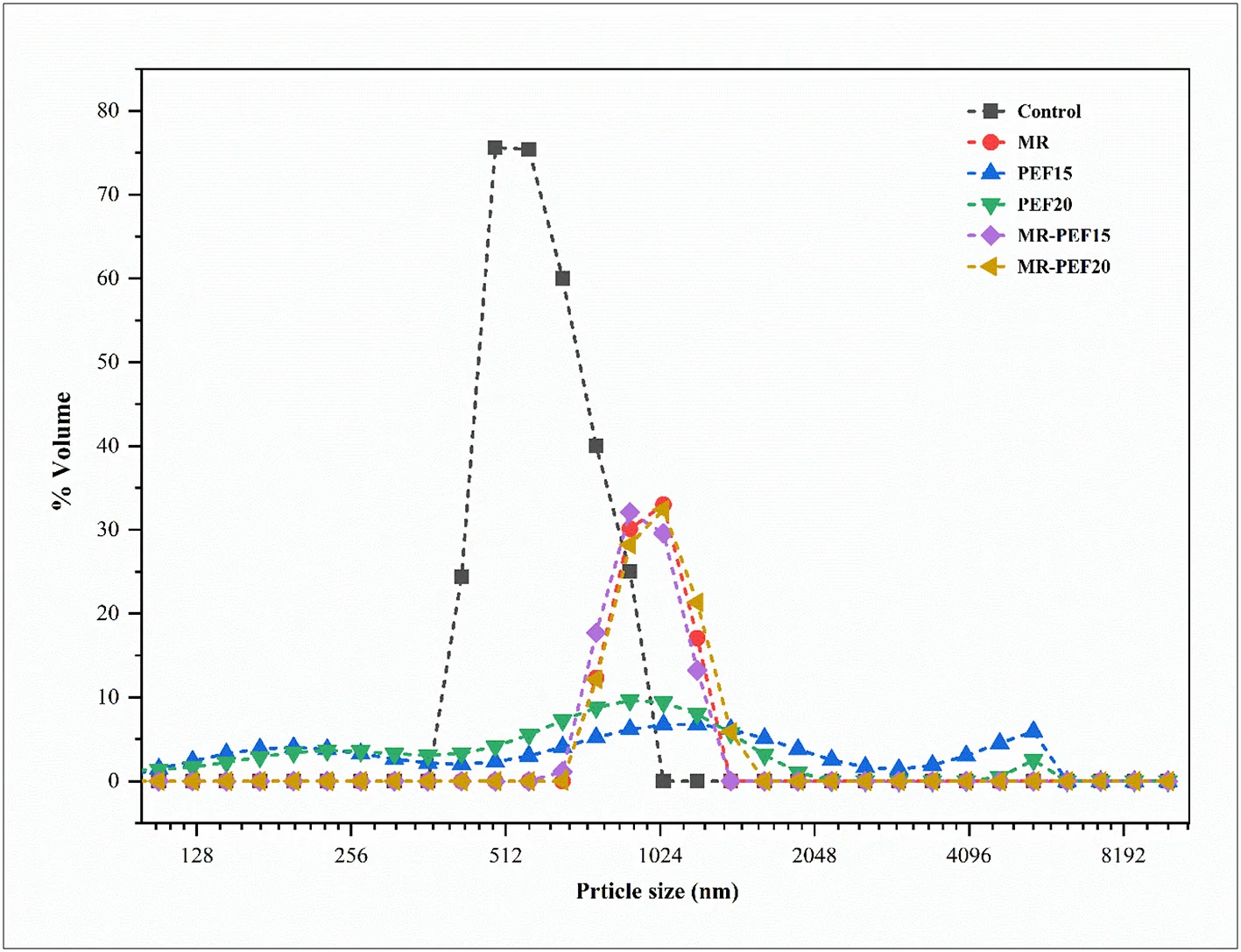
Volume-based particle size distribution (PSD) profiles of RPSPI after MR, PEF, and combined MR-PEF treatments. The PSD profiles represent the distribution of hydrated particle populations measured by dynamic light scattering (DLS), while the corresponding Z-average hydrodynamic diameter and polydispersity index (PDI) values are summarized in Table 1. C, Control; MR, Micronization; PEF15, 15 kV/cm; PEF20, 20 kV/cm; MR-PEF15, MR+15kV/cm; MR-PEF20, MR+20 kV/cm.

**Table 1:** Impact of combined MR-PEF on the Z-average hydrodynamic diameter, polydispersity index (PDI) and zeta potential of PSPI.

Treatments	Particle size (nm)	PDI	Zeta potential (mV)
Control	2,144 ± 18^a^	0.7021 ± 0.02^a^	−56.75 ± 0.12^a^
MR	1,771 ± 13^b^	0.6543 ± 0.04^b^	−54.63 ± 0.08^b^
PEF15	1,523 ± 09^c^	0.6012 ± 0.02^c^	−51.47 ± 0.06^c^
PEF20	1,376 ± 08^d^	0.5813 ± 0.01^d^	−48.42 ± 0.07^d^
MR-PEF15	521.7 ± 06^e^	0.4587 ± 0.03^e^	−44.34 ± 0.04^f^
MR-PEF20	547.7 ± 07^f^	0.4675 ± 0.02^e^	−46.24 ± 0.05^e^

Different letters (a–f) show significant differences (*p* < 0.05) among treatments in the same column. C, Control; MR, Micronization; PEF15, 15 kV/cm; PEF20, 20 kV/cm; MR-PEF15, MR+15kV/cm; MR-PEF20, MR+20 kV/cm.

The polydispersity index (PDI) was evaluated to determine the width of the particle size distribution and dispersion heterogeneity, and the results are listed in Table 1. The findings indicated that the control sample demonstrated the highest PDI value (0.7021), suggesting a large particle-size distribution and high heterogeneity within the native protein dispersion. However, after treatment with MR and PEF, the PDI values decreased to 0.6543 (MR), 0.6012 (PEF15), and 0.5813 (PEF20), indicating reduced particle-size heterogeneity. The combined treatments (MR-PEF) showed the lowest PDI values at 0.4587 for MR-PEF15 and 0.4675 for MR-PEF20, suggesting increased dispersion characteristics compared with the control and individually treated samples. Even though the PDI values remained above 0.3 for all samples, demonstrating the occurrence of numerous particle populations and moderate polydispersity, the progressive reduction compared with the control confirms improved dispersion homogeneity after treatment. Thus, the PDI findings support the decrease in DLS-derived particle size and indicate reduced heterogeneity rather than complete particle uniformity.

The individual treatment of MR reduced particle size through mechanical disruption of the compact protein matrix, producing smaller particles with improved surface activity. Nonetheless, the newly exposed functional groups may increase intermolecular interactions during hydration, resulting in partial reassociation and formation of hydrated clusters. Likewise, PEF treatment can change protein structure by reducing non-covalent connections, inducing molecular rearrangement, and exposing functional groups. These structural changes may widen the PSD profile despite reducing the average hydrodynamic diameter. On the other hand, enhancing the electric field strength from PEF15 to PEF20 further increased molecular mobility and particle interactions, contributing to changes in particle distribution (19).

Moreover, the combined MR-PEF treatments delivered a significant reduction in average hydrodynamic diameter while sustaining narrower PSD profiles compared with individual treatments, demonstrating enhanced dispersion characteristics. The individual treatment of MR likely disrupted large protein aggregates and improved accessibility of internal structures; however, following PEF treatment accelerated controlled rearrangement of unfolded protein chains. Therefore, MR-PEF treatment produced smaller protein particles with lowered size heterogeneity and enhanced dispersion behavior, indicating a combined effect between mechanical fragmentation and electric-field-induced structural modification.

Generally, the DLS, PSD and PDI findings provide complementary facts about the structural changes brought by PEF and MR treatments. The decrease in DLS particle size proves the breakdown of large native aggregates, while the broader PSD profiles reflect the coexistence of smaller particles and hydrated aggregate populations. The decreased PDI values further demonstrate reduced particle-size heterogeneity after treatment. Thus, the observed PSD changes should be interpreted as a balance between particle fragmentation and controlled molecular reassociation rather than simple particle enlargement (20). These structural modifications may contribute to improved hydration capacity, interfacial activity, and functional properties of RPSPI (21).

### 3.2 Particle size

Table 1 represents the Z-average hydrodynamic diameter of RPSPI samples after MR, PEF, and combined MR-PEF treatment. The findings indicated that the treatment significantly (*p* < 0.05) reduced the average particle size of all treated samples compared to control samples. The findings demonstrated that the Z-average hydrodynamic diameter reduced from 2,144 nm in the control sample to 1,771 nm after MR treatment, demonstrating that micronization efficiently disrupted large native protein aggregates. Mechanistically, the mechanical shear and high collision forces generated during micronization reduce particle size, break down protein aggregates into smaller, more uniform particles, expose carboxyl and amino acid groups within protein aggregates, and enhance the specific surface area. Similarly, studies reported that MR reduced particle size in quinoa protein isolates, leading to mechanical dissociation of aggregates, electrostatic repulsion, and partial protein unfolding (22). The PEF treatment showed a significant reduction in Z-average diameter compared to the control and MR-treated samples, with 1,523 nm and 1,376 nm, followed by PEF15 and PEF20, respectively. The reduction in Z-average diameter can be attributed to electroporation effects and disruption of intermolecular interactions within protein aggregates. The applied electric field induces molecular polarization and structural stress, which can weaken non-covalent interactions, including hydrogen bonding, hydrophobic interactions, and electrostatic forces, resulting in partial unfolding and dissociation of protein aggregates into smaller particles (19). Our results align with previous studies reporting that PEF treatment reduced the particle size of protein isolates compared with untreated samples (23, 24).

Furthermore, the combined treatments of MR-PEF delivered the best reduction in particle size, with Z-average hydrodynamic diameters of 521.7 nm for MR-PEF15 and 547.7 nm for MR-PEF20. The combined effect of mechanical fragmentation by MR and structural modification induced by PEF resulted in more efficient disruption of large protein aggregates compared with individual treatments. Furthermore, the reduced PDI values observed after combined treatments (0.4587 for MR-PEF15 and 0.4675 for MR-PEF20) suggest reduced particle-size heterogeneity and improved dispersion characteristics compared with the control sample. Notably, the particle size values listed in Table 1 correspond to the Z-average hydrodynamic diameter measured by DLS. In contrast, the PSD curves in Figure 1 correspond to the volume-based distribution of hydrated particles. The occurrence of large particles in the PSD profiles after treatment does not conflict with the reduction in average particle size. These findings indicate that MR and PEF treatments disrupted large aggregates and simultaneously facilitated the controlled association of hydrated particles.

### 3.3 Zeta-potential

The zeta potential of RPSPI after MR, PEF, and their combined treatment effects are presented in Table 1. The zeta potential value of all treated samples remains negative, indicating that the RPSPI particles had a negative surface charge during the treatment. The main reason for this negative charge is the ionization of surface acidic amino acid residues on the surface of the protein (25). However, the magnitude of the zeta potential decreased in all individual and combined treatments, even though the sign of the negative charge was constant. The control samples showed the highest negative zeta potential (−56.75 mV), indicating strong electrostatic repulsion among the particles and increased colloidal stability, while the values reduced significantly in treated samples. The reduction in negative charges during MR (−54.63 mV) could be attributed to the distraction of the native structure and reorientation of charge groups. The mechanical treatment can expose hydrophobic areas or hide some ionizable residues within the new form aggregates, leading to a decrease in surface negative charges (26). On the other hand, the samples treated with 15 kV/cm and 20 kV/cm also showed significant reductions in zeta potential (−52.47 mV and −48.42 mV), indicating that the PEF treatment modified the protein surface. The high electric field strength after 20 kV/cm may promote greater surface neutralization or chemical rearrangements, leading to an effective reduction in negative charges. The lowest value was observed in the combined treated samples (−44.34 mV) followed by MR-PEF15. The combined treatment of MR and PEF showed the lowest zeta potential values among the treated samples, indicating that the effective structural modification was achieved through mechanical and electrical processing. These findings are consistent with the PSD results, as the treated samples showed a higher PS distribution. This might be because the aggregate formation in aqueous solution is readily disrupted by electrostatic repulsion, which creates an energy barrier to particle aggregation (27). Since it promotes cohesive interactions, which are essential for gelation and emulsification, a reduction in surface charge is advantageous in many applications. Therefore, changes in zeta potential should be viewed as revealing functional reformation rather than simple instability.

### 3.4 Intrinsic fluorescence analysis

Figure 2 shows intrinsic fluorescence results obtained by intrinsic fluorescence spectroscopy, indicating tertiary structural modification in RPSPI after MR, PEF, and combined treatments. The outcomes indicated that the emission spectra of all treated samples were comparable, with a maximum emission wavelength of around 335 to 340 nm, primarily due to tryptophan residues. The change in intensity indicates structural changes in the protein since tryptophan fluorescence is extremely sensitive to its surroundings (28).

**Figure 2:**
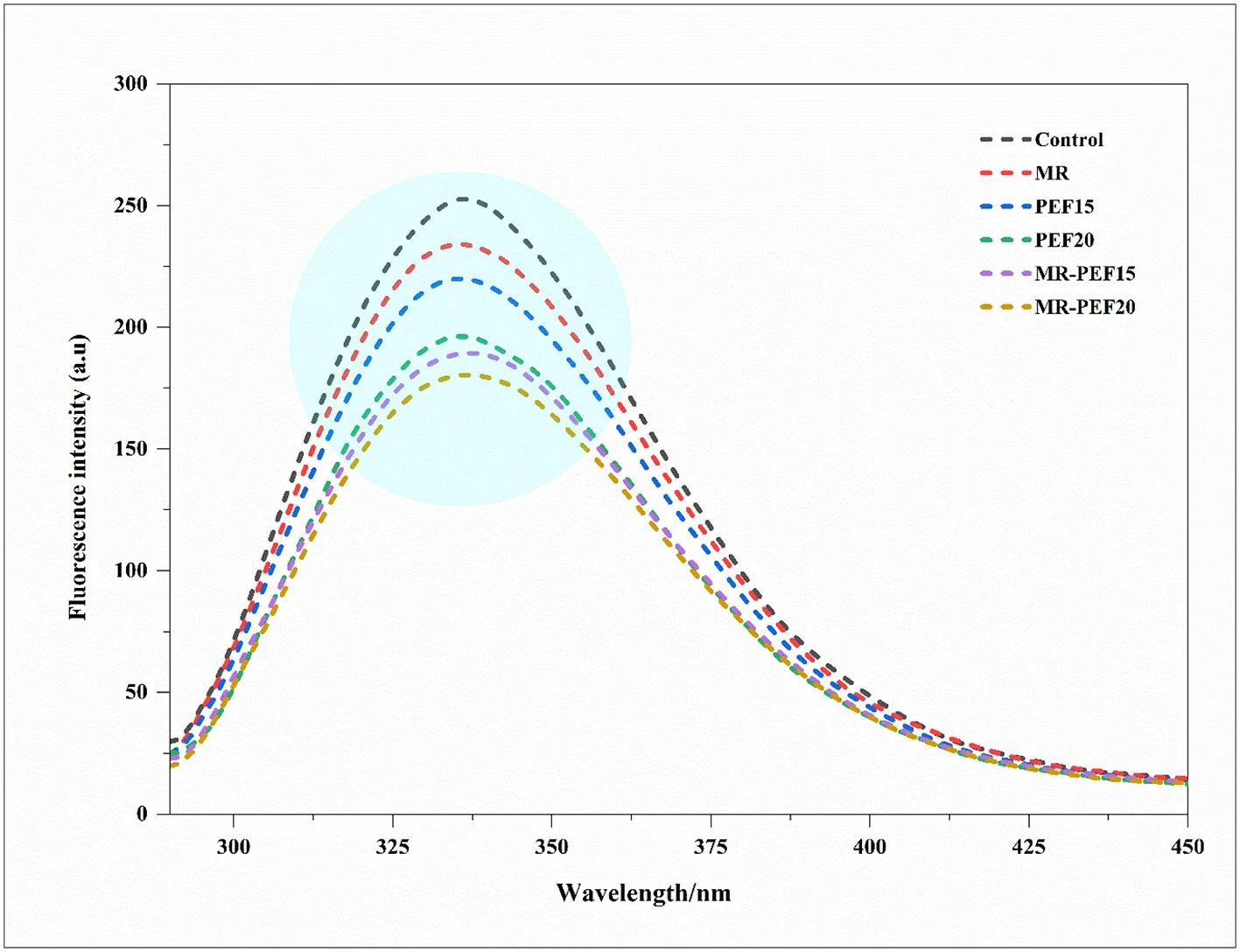
Fluorescence Intensity of RPSPI after MR, PEF and their combined treatmen; C, Control; MR, Micronization; PEF15, 15 kV/cm; PEF20, 20 kV/cm; MR-PEF15, MR+15kV/cm; MR-PEF20, MR+20 kV/cm.

The control samples showed the highest fluorescence intensity (≈250 a.u.) compared to all treated MR, PEF, and combined treatment samples, which exhibited a reduction in fluorescence intensity. The gradual reduction in intensity demonstrates that both electrical and mechanical processing changed the protein's tertiary structure. The samples treated with MR showed a slight decrease in intensity, indicating partial unfolding induced by collisions, impacts, and shear force during MR treatment (29). These disruptions could expose previously buried tryptophan residues to the aqueous environment, resulting in a reduction in fluorescence intensity (20). Similarly, the samples treated with PEF induced a higher decrease in intensity, particularly at 20 kV/cm. On the other hand, the fluorescence emission spectra of all treated samples showed a highest emission wavelength of approximately 335–340 nm indicating that the overall emission position remained relatively unchanged after treatment. The fluorescence emission was primarily related to the intrinsic fluorescence of tryptophan residues, which are highly sensitive to changes in their surrounding environment. The reduction in fluorescence intensity indicates fluorescence quenching and suggests that the treatment altered the microenvironment and accessibility of tryptophan residues with in the protein structure. These modifications may be attributed to protein unfolding, conformational rearrangement or altered protein -protein interactions, which can modify the surrounding environment of the fluorophores and consequently their fluorescence behavior. Therefore, the observed reduction in fluorescence intensity provides evidence that MR and PEF treatment induced changes in the tertiary structure of RPSPI. As a result, aromatic residues are more exposed to polar solvents, which reduces fluorescence intensity (30). The effectiveness of increasing the electrical strength to 20 kV/cm suggests a significant structural modification.

On the other hand, the combined treatment of MR and PEF showed the lowest intensity, followed by MR-PEF20, showing the highest drop, indicating the effectiveness of MR and PEF. Initially, the MR loosens the compact protein matrix by increasing the molecule's vulnerability to further electric field exposure. At the same time, PEF partially damages the protein structure, resulting in increased exposure of aromatic residues. The treatment primarily altered fluorescence intensity, rather than residual polarity, as no significant shift in λmax was observed among the samples (31). Zhang et al. (32) reported that non-thermal processing of proteins commonly causes minor changes in tertiary structure, exposing aromatic residues as evidenced by changes in fluorescence intensity, but the peak emission wavelength (λmax) remains constant. As a result, the protein maintained its tertiary structure, rather than fully denaturing. In addition, the fluorescence data are consistent with the zeta potential and PS distribution, indicating that protein unfolding and intermolecular reassociation occur following treatment. On the other hand, intrinsic fluorescence results showed that the combined treatment of MR and PEF effectively changed the tertiary structure of RPSPI. Finally, such related unfolding may improve functional properties and surface activity, thereby promoting the application of these green methods for value-added utilization of plant protein.

### 3.5 Secondary structure: FTIR analysis

The structural modifications in RPSPI induced by MR, PEF, and their combined treatments, MR-PEF, were analyzed using FTIR spectroscopy (Figure 3A). The findings indicated that all samples show protein absorption bands, such as Amide I, Amide II and Amide III regions. Figure 3A, demonstrates that the Amide I band around 1,600–1,700 cm^−1^ is generally associated with C=O stretching vibration of peptide bonds, which is specifically sensitive to alterations in protein secondary structure. Similarly, 1,500–1,600 cm^−1^ indicates the Amide II band, mainly associated with N-H bending and C-N stretching vibrations, and 1,200–1,300 cm^−1^ indicates Amide III regions associated with N-H deformation and C-N stretching, indicating the structural changes of RPSPI after treatment. The findings also demonstrate that, as compared to control samples, MR, PEF and MR-PEF treatments visibly change the intensity and profile of the Amide I region (11, 33). These modifications in spectral vibration demonstrate that the treatments change the protein conformational rearrangement and molecular environment. However, the direct interpretation of peak changes alone cannot quantitatively determine the variations in individual β-sheet, α-helix, β-turn, and random coil structures because the Amide I region comprises overlapping contributions from diverse secondary structural elements (34, 35). Therefore, the Amide I region was further subjected to peak deconvolution for quantitative estimation of the relative contribution of various secondary structures and the calculated secondary structure distributions are listed in Table 2. The results demonstrate that the control sample revealed a dominant β-sheet structure (58.2 ± 0.12%), followed by random coil (19.3 ± 0.09%), β-turn (12.3 ± 0.09%), and α-helix (10.3 ± 0.11%) structures. The high percentage of β-sheets indicates that the native RPSPI acquires comparatively disciplined molecular arrangements stabilized by intermolecular hydrogen bonding and hydrophobic interactions.

**Figure 3:**
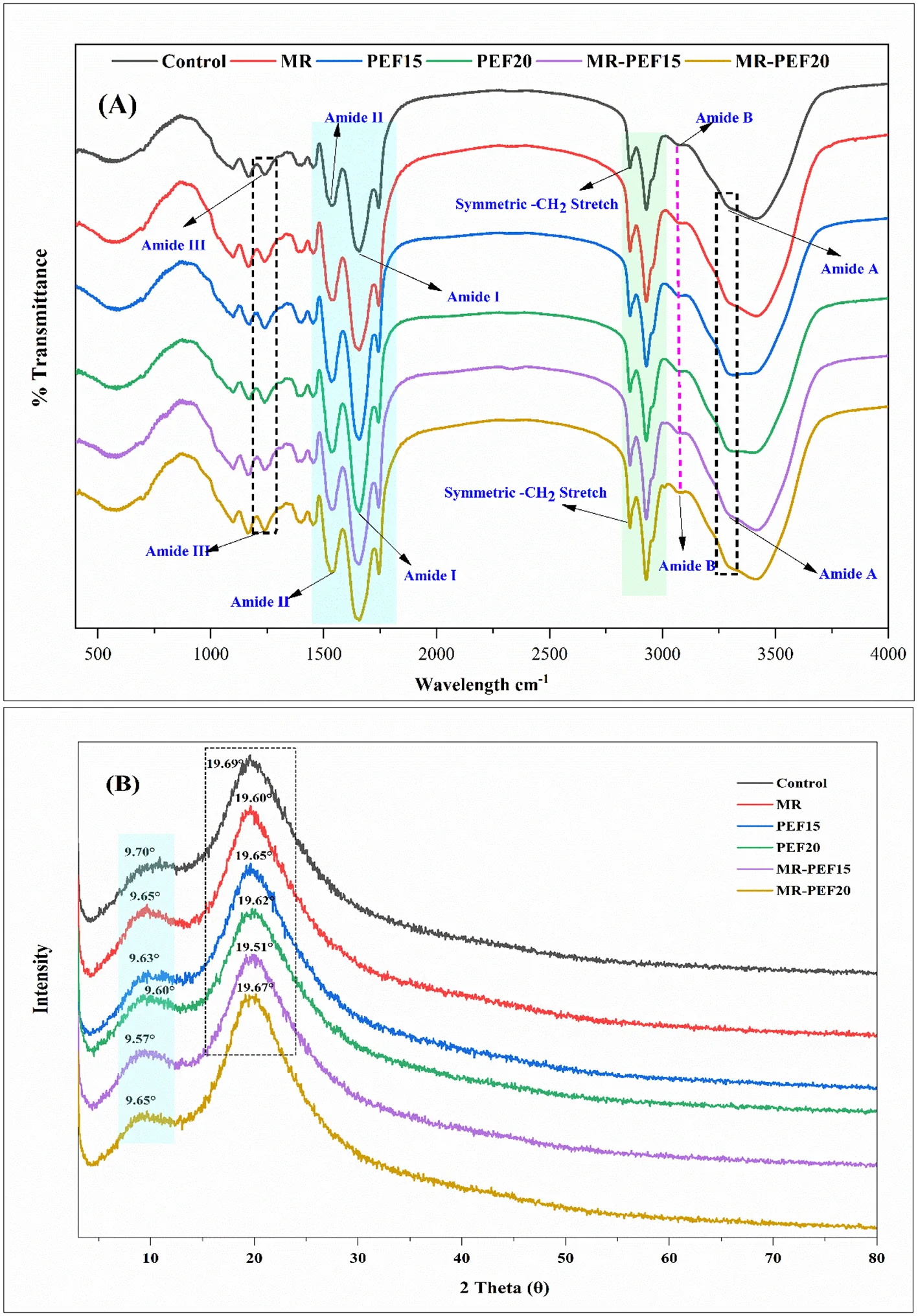
FTIR spectra **(A)**, and XRD **(B)** of RPSPI after MR, PEF and their combined treatmen; C, Control; MR, Micronization; PEF15, 15 kV/cm; PEF20, 20 kV/cm; MR-PEF15, MR+15kV/cm; MR-PEF20, MR+20 kV/cm.

**Table 2:** Relative contents of secondary structures in RPSPI determined by Amide I deconvolution.

Treatment	α-helix (%)	β-sheet (%)	β-turn (%)	Random coil (%)
Control	10.3 ± 0.09^a^	58.2 ± 0.12^c^	12.3 ± 0.11^bc^	19.3 ± 0.09^c^
MR	10.8 ± 0.10^a^	62.3 ± 0.15^a^	13.4 ± 0.14^b^	13.5 ± 0.08^d^
PEF15	1.1 ± 0.02^d^	22.8 ± 0.14^e^	22.2 ± 0.16^a^	53.8 ± 0.06^a^
PEF20	4.2 ± 0.03^c^	33.8 ± 0.17^d^	14.4 ± 0.08^b^	47.7 ± 0.05^b^
MR-PEF15	7.4 ± 0.05^b^	60.9 ± 0.19^b^	12.0 ± 0.07^c^	19.6 ± 0.07^c^
MR-PEF20	9.9 ± 0.08^ab^	63.9 ± 0.21^a^	11.5 ± 0.09^cd^	14.7 ± 0.05^d^

Different letters (a–e) show significant differences (*p* < 0.05) among treatments in the same column. C, Control; MR, Micronization; PEF15, 15 kV/cm; PEF20, 20 kV/cm; MR-PEF15, MR+15kV/cm; MR-PEF20, MR+20 kV/cm.

Furthermore, MR treatment shows that the β-sheet percentage enhanced from 58.2 ± 0.12% to 62.3 ± 0.15%, while random coil structures reduced from 19.3 ± 0.09% to 13.50.08%, and the α-helix and β-turn contents showed slight variations as 10.8 ± 0.10% and 13.40.14%, respectively. These outcomes indicated that MR mainly promoted rearrangement rather than massive unfolding. Further, the mechanical shear forces generated during MR interrupted large protein aggregates and exposed internal protein regions, which may subsequently undergo intermolecular interactions and rearrangement into more ordered structures. PEF treatment showed more visible changes in secondary structure composition as compared to MR, showing that PEF15 treatment resulted in a significant rearrangement of secondary structures, with β-sheet content reducing to 22.8 ± 014% and random coil content increasing to 53.8 ± 0.06%. Likewise, treatment PEF20 showed increased random coil content (47.7 ± 0.05%) with partial recovery of β-sheet structures (33.8 ± 017%). These findings demonstrate that PEF accelerated impressive conformational flexibility by disturbing the native ordered protein arrangement. The applied electrical stress weakened non-covalent interactions, including electrostatic interactions, hydrogen bonding, and hydrophobic associations, resulting in partial unfolding and alteration of well-ordered structures into more flexible conformations (26).

On the other hand, as compared to MR and PEF treatment alone, the combined treatment MR-PEF demonstrated various structural responses. The results indicated that MR-PEF15 and MR-PEF20 showed higher β-sheet contents was 60.9 ± 0.19% and 63.9 ± 0.21%, respectively, followed by lower random coil contents as 19.6 ± 0.07% and 14.7 ± 0.05%, α-helix contents were 7.4 ± 0.05% and 9.9 ± 0.08%, while β-turn structures accounted for 12.0 ± 0.07% and 11.5 ± 0.09%, respectively. These findings demonstrate that combined treatment promoted partial structural recovery and molecular reordering following initial disruption. The MR treatment likely fragmented large protein aggregates and improved molecular availability, while subsequent PEF exposure accelerated rearrangement of unfolded protein chains (29). Moreover, the FTIR findings are consistent with PDI analyses and particle size. The decline in hydrodynamic diameter and PDI after MR-PEF treatment shows efficient disruption of larger aggregates and enhanced dispersion properties. The regaining of organized β-sheet structures after combined treatment indicates that protein molecules underwent controlled reorganization rather than random structural degradation (19). Therefore, PEF and MR treatments regulated RPSPI structure through a balance between conformational modification and molecular reassociation and aggregate disruption. In general, the FTIR spectra (Figure 3A) together with quantitative Amide I deconvolution (Table 2) reveal that MR and PEF treatments caused treatment-dependent conformational transformations in RPSPI proteins. The MR generally promoted structural rearrangement, whereas PEF triggered remarkable disruption of ordered structures and improved conformational flexibility. Finally, the combined treatment MR-PEF integrated these effects by promoting both aggregate fragmentation and controlled protein reorganization (18).

### 3.6 XRD

The crystalline structure of the RPSPI sample treated with MR, PEF, or their combined treatment was evaluated by XRD (Figure 3B). The outcomes revealed that all treated samples exhibit two peaks in the XRD spectra near 2θ = ~20° and ~10°, which are referred to as crystalline areas I and II, suggesting ordered protein structural domains (36). The sample treated with MR showed a slight shift in the higher-angle peak, ranging from ~19.69° to ~19.60°, while the lower-angle peak changed from ~9.70° to ~9.65°. The PEF-treated samples exhibited a higher-angle peak at ~19.65°, and a lower-angle peaks at ~9.63°, respectively. The combined treatment MR-PEF15 showed significant shifts at lower and higher angles up to ~19.51° and ~9.57°, respectively. However, the diffraction peaks indicated that native RPS protein isolates exhibit an aggregated or semi-aggregated structure, consistent with earlier protein XRD results that associate residual order with functionality (37). The gradual decrease in intensity indicates that MR breaks the hydrophobic interaction and the H bond, resulting in a significant decrease in relative crystallinity.

Additionally, following PEF treatment, decreases in higher and lower-angle intensities indicate a partial disruption of the crystalline structure and a reduction in the relative crystalline size of the sample. Mechanistically, PEF treatment at high electric energy disrupts the molecular arrangement within the crystalline area, resulting in a shift toward a more amorphous structure. This behavior may be associated with weakening the intermolecular hydrogen bonds and molecular rearrangement. A significant reduction was observed in the combined MR-PEF15 treatment samples, indicating that the mechanical and electrical impact of MR and PEF significantly altered the intensity. In addition, the electroporation induced by PEF treatment might induce structural rearrangement and granule damage, resulting in lower diffraction intensity and reduced structural perfection. Similarly, previous studies reported that PEF treatment reduces the reduction in lower and higher-angle intensities in XRD results across various PEF-treated starch sources (38). Moreover, the decrease in peak intensity and changes in diffraction pattern indicate changes in the ordered structural organization of RPSPI following treatment. These modifications suggest a reduction in the degree of structural ordering and crystallinity of the protein matrix, which may be attributed to treatment induced structural rearrangement and disruption of ordered changes.

### 3.7 SEM

Figure 4 shows SEM images of MR, PEF, and their combined effect on RPSPI, demonstrating treatment-induced morphological changes, including disruption of compact aggregates, increased surface irregularity, and differences in porosity and particle organization compared with the control sample. Figure 4 shows that the control samples had a larger, more compact, aggregated, and irregular structure compared to the treated samples. On the other hand, MR, PEF, and combined treatment significantly alter these compact aggregates, showing smaller, smoother, and fractured particles with enhanced surface porosity. However, alterations in the microstructure are linked to emulsifying properties, solubility, foaming, and particle size reduction, all of which are consistent with our results.

**Figure 4:**
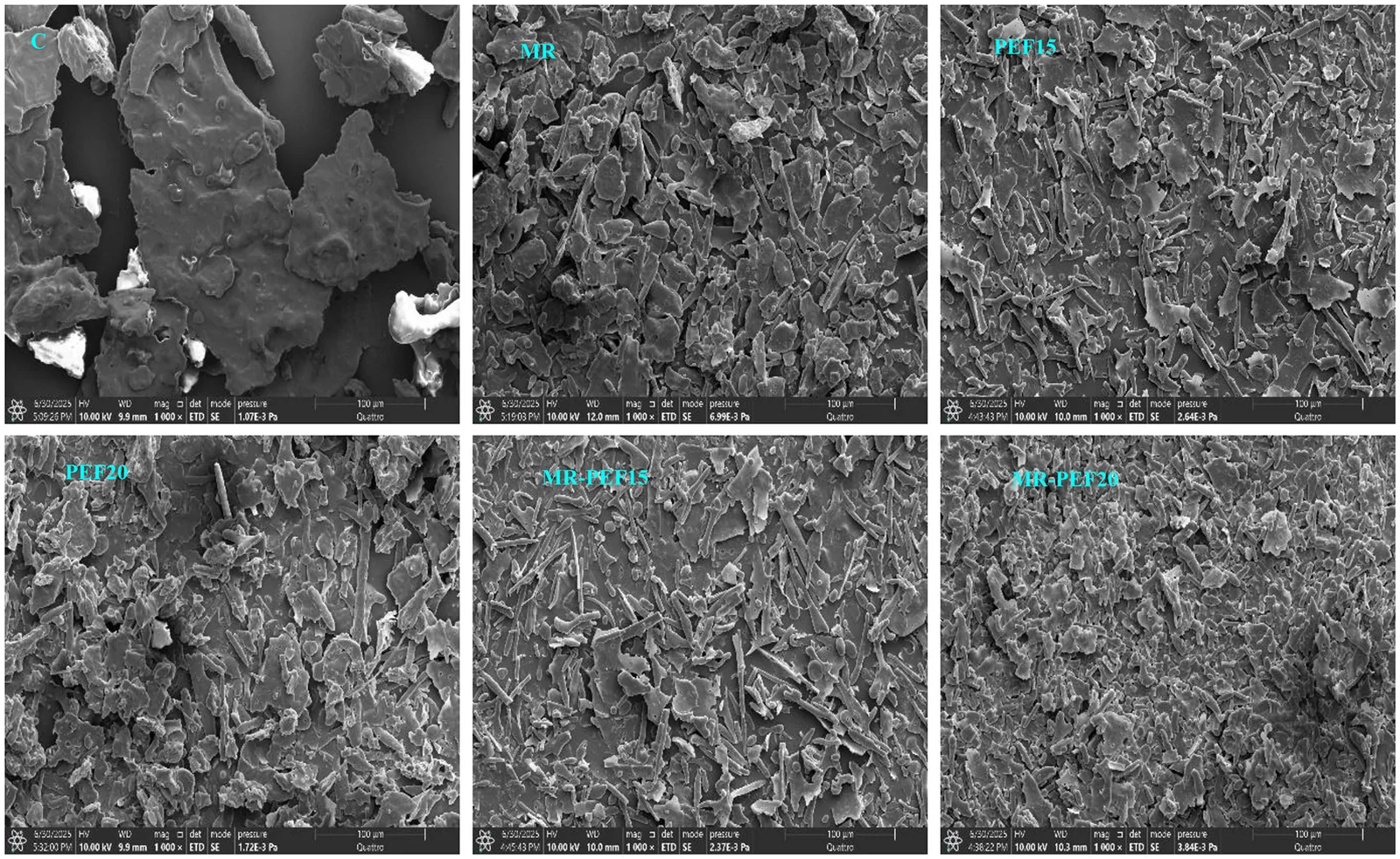
SEM illustration of RPSPI after MR, PEF and their combined treatmen; C, Control; MR, Micronization; PEF15, 15 kV/cm; PEF20, 20 kV/cm; MR-PEF15, MR+15kV/cm; MR-PEF20, MR+20 kV/cm.

The SEM images, presented with improved quality in the revised manuscript, more clearly revealed treatment-induced morphological changes, including disruption of compact aggregates, increased surface irregularity, and differences in porosity and particle organization compared with the control sample.

In addition, during processing, the partial unfolding of protein molecules and the loosening of compact aggregates led to structural alteration. Mechanistically, the reduction in particle size during MR processing loosened the RPSPI structural arrangements, which might be driven by mechanical and shear forces during treatment, thereby modifying the globular structure by preventing aggregation. Our results agree with similar findings reported after MR processing of red pepper seed (29), soy (12), and sorghum protein isolates (11). Additionally, PEF-treated RPSPI samples exhibited porous, smaller pieces and a shattered surface, indicating that PEF treatment significantly altered RPSPI's structure. Similar results have been reported after PEF processing of myofibrillar protein and quinoa protein isolates (23, 24). Further, the combined effect of MR and PEF, such as MR-PEF15 and MR-PEF20, showed more significant effects on structural alterations, indicating reduced surface smoothness and a greater number of finer particles, which are linked to reduced foaming properties and to particle size associated with protein linkage through disulfide or hydrophobic interactions. Mechanistically, the electrophoresis effect of PEF and the mechanical force of MR enhanced the number of protein surface and altered the microstructure of RPSPI. Furthermore, the FTIR results are consistent with the SEM results, demonstrating that an enhancement in β-sheet and β-turn, caused the structural modification. Previous studies revealed that partial denaturation of proteins results in a greater number of flexible molecular arrangements, which help create a finer and more porous microstructure, followed by increasing in β-sheet (39).

### 3.8 Thermal properties

The thermal properties of MR and PEF-treated RPSPI samples were analyzed using differential scanning colorimetry. Table 3 represents the impact of MR, PEF, and combined treatment effect on the thermal properties of treated RPSPI. The findings revealed slight changes in the denaturation temperature (*T*_d_) and enthalpy change (ΔH) following MR and PEF treatment. The control sample showed the thermal properties of RPSPI to be 49.03 °C, 84.62 °C, and 103.22 J/g for *T*_0_, *T*_d_, and ΔH, respectively. After MR treatment, a minimal change was observed in *T*_0_, *T*_d_, and ΔH values as 50.51 °C, 90.32 °C, and 111.07 J/g. The MR treatment caused substantial cavitation, shear force, and variability in protein structure, disrupting hydrophobic contacts, weak disulfides, and hydrogen bonds, resulting in unfolding, reduced compactness, and smaller protein aggregates (40, 41). Our findings agree with Thakur, Sharma (11), who observed a slight enhancement in ΔH and *T*_d_ values after homogenization treatments, attributed to alterations in tertiary structure, disintegration of the linkage, and the formation of smaller and less organized aggregates.

**Table 3:** Impact of combined MR-PEF on the thermal properties of PSPI.

Treatments	Onset temperature *T*_o_ (°C)	Denaturation temperature *T*_d_ (°C)	Enthalpy change ΔH (J/g)
Control	49.03 ± 0.12^bc^	84.62 ± 0.13^d^	103.22 ± 0.23^d^
MR	50.51 ± 0.14^b^	90.32 ± 0.15^a^	111.07 ± 0.24^a^
PEF15	55.51 ± 0.15^a^	88.37 ± 0.19^bc^	109.25 ± 0.19^b^
PEF20	49.75 ± 0.10^bc^	86.75 ± 0.14^c^	107.83 ± 0.17^c^
MR-PEF15	51.53 ± 0.09^b^	89.65 ± 0.16^b^	110.78 ± 0.29^ab^
MR-PEF20	49.33 ± 0.07^bc^	88.62 ± 0.17^bc^	108.52 ± 0.27^bc^

Different letters (a–d) show significant differences (*p* < 0.05) among treatments in the same column. C, Control; MR, Micronization; PEF15, 15 kV/cm; PEF20, 20 kV/cm; MR-PEF15, MR+15kV/cm; MR-PEF20, MR+20 kV/cm.

In addition, the PEF treatment showed a minor increase in the *T*_0_, *T*_d_, and ΔH values of 49.75 °C, 86.37 °C, and 107.83 J/g, respectively. The moderate or slight enhancement showed better molecular connectivity and increased thermal stability within the protein structure, resulting in RPSPI treated with PEF being more resilient to thermal denaturation during PEF processing. This indication showed that PEF-induced RPSPI conformational changes, such as partial unfolding and molecular rearrangements, which promote the formation of stronger intermolecular connections, including electrostatic interactions, hydrophobic association, and hydrogen bonding, might be responsible for this behavior (42). Our results are consistent with the findings of Zhang et al. (24), who recently reported that PEF treatment effected the *T*_d_ of quinoa protein isolates, indicating that some PEF treated samples exhibited higher *T*_d_ values than untreated samples. These changes were attributed to PEF induced modifications in protein structure. Similarly, the changes in *T*_d_ observed in the present study suggest that MR assisted PEF treatment altered the thermal stability and structural organization of RPSPI. However, the effect of PEF treatment on *T*_d_ may depend on the processing conditions, including pulse number and treatment intensity.

Furthermore, the combined treatment of MR and PEF also showed a slight increase in *T*_0_, *T*_d_, and ΔH, to 51.53 °C, 89.65 °C, and 110.78 J/g, respectively, followed by sample MR-PEF15. However, there was no significant difference in *T*_0_, *T*_d_, and ΔH values between the MR-PEF20 and the control samples, indicating that enhanced PEF treatment slightly increased the thermal properties. Additionally, the slight increase in ΔH values observed with enhanced PEF voltage in the combined treatment might indicate that the protein networks were partially stabilized or reaggregated, resulting in the formation of tiny aggregates via intermolecular cross-links that slightly enhanced the thermal energy. Previous studies have shown that variations in the thermal properties of PEF might affect protein thermal stability under different PEF processing conditions (43). In addition, the thermal properties of the RPSPI provide important insights into the structural stability of the RPSPI, which defines its thermal transition behavior and could be used to maximize its functional characteristics and possible application in food systems.

### 3.9 Turbidity and solubility

Figure 5A represents the effect of MR, PEF, and their combined treatment impact on turbidity and solubility of RPPSI. The results indicated that the turbidity of treated samples was significantly reduced, while the solubility was significantly enhanced. The control samples showed a turbidity value of 0.80 and the solubility of 39.4%, indicating poor solubility and the presence of larger particles. The samples treated with MR showed significantly reduced turbidity of the RPSPI solution to 0.77, while increasing solubility to 42.2%. Mechanistically, MR treatment disrupts protein aggregates through high shear, enhancing flexibility, reducing particle size, and ultimately reducing turbidity and increasing solubility of the treated RPSPI solution. On the other hand, MR might be exposed to polar/charged residues, causing effective electrostatic repulsion due to the high negative charge (zeta potential), which stops reaggregation and provides a stable solution. Our findings correspond with those of Manzoor et al. (29), who reported that high-shear micronization treatment significantly increased solubility and decreased turbidity % of RPSPI.

**Figure 5:**
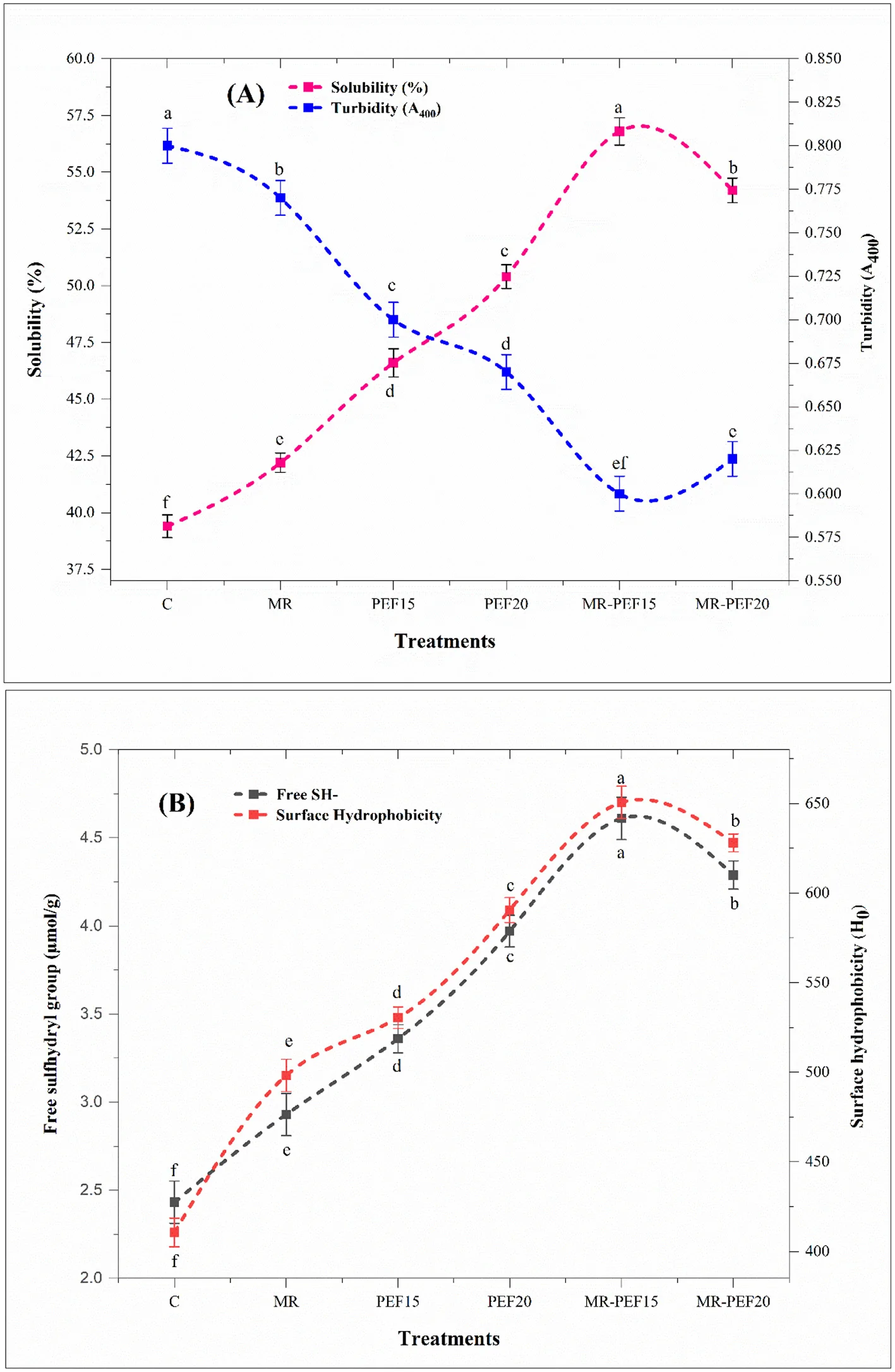
Turbidity and Solubility **(A)**, and Free SH^−^ and *H*_0_ **(B)** of RPSPI after MR, PEF and their combined treatmen; C, Control; MR, Micronization; PEF15, 15 kV/cm; PEF20, 20 kV/cm; MR-PEF15, MR+15kV/cm; MR-PEF20, MR+20 kV/cm; while the different letters (a-f) above the bars show the significant difference between treatments and control (Tukey's HSD, *p* < 0.05).

Similarly, PEF treatment alone significantly increased solubility to 50.4% and reduced turbidity to 0.67%, compared with control samples, as shown in Figure 5A. Previously, studies revealed that PEF treatment mechanistically polarized, thereby enhancing solubility by revealing polar groups and partially unfolding the protein structure. Furthermore, contact between proteins and water is enhanced by electrostatic interactions and non-covalent bonds formed with protein molecules driven by free radicals produced by PEF (16). Another study also reported that the breakdown of protein particles and covalent bonds, such as hydrogen bonds and hydrophobic bonds, by PEF might be caused by the increase in protein solubility with reducing the protein particle size while preventing protein aggregation and improving protein solubility (44). Our results agree with Wang et al. (19) and Zhang et al. (45), who reported that the solubility of soybean and canola protein isolates was significantly enhanced after PEF treatment compared to untreated samples. Additionally, the reduction in turbidity % could be attributed to electrostatic repulsion and the electric field force, which could reduce the quantity of light scattering and particle size of soluble protein aggregates. The findings agree with Wang et al. (19) who reported that, compared with untreated samples the turbidity% of soy protein were reduced after PEF treatment.

Figure 5A shows the combined treatment of MR and PEF, which resulted in a significant reduction in turbidity to 0.6% and an increase in solubility to 56.8%, compared to the control samples. Mechanistically, MR treatment produced strong shear forces and cavitation. which breaks large protein aggregates into smaller particles, thereby increasing surface area by exposing hydrophilic groups to water. On the other hand, PEF breaks hydrogen bonds and hydrophobic interactions, leading to partial protein unfolding and improved dispersion in water. Finally, these structural modifications increased protein-water interactions while reducing the formation of insoluble protein aggregates, resulting in uniform, smaller, more dispersed particles that scattered less light, causing lower turbidity and enhanced solubility. Generally, the combined MR and PEF treatment effectively enhanced the functional properties of RPSPI by promoting a more dispersed and hydrated protein structure.

### 3.10 Free SH^−^ and surface hydrophobicity (*H*_0_)

The effects of MR, PEF, and the combined treatment on SH^−^ and *H*_0_ are shown in Figure 5B. The free SH^−^ of RPSPI is a significant indicator of protein structural change, revealing the breakdown of the disulfide bond after PEF and MR treatment, and their combination treatment by exposing sulfur-containing amino acids. The findings show that SH^−^ content increased to 2.93 μmol/g and 3.97 μmol/g following MR and PEF treatment, respectively, while the combined treatment MR-PEF15 (4.61 μmol/g) showed significantly higher SH^−^ content, indicating that the combined treatment effectively enhanced the SH^−^ group. Mechanistically, the shear force and cavitation produced during treatment disrupted the compact protein structure and reduced particle size, which might also have led to an increase in SH^−^ content. The mechanical impact of the MR partially unfolded protein molecules, revealing previously hidden SH^−^ groups that were unreachable inside the native protein structure.

Additionally, the enhanced SH^−^ of RPSPI may have resulted from the disintegration of protein aggregates and partial breaking of weak disulfide bonds (-S-S-). On the other hand, PEF treatment unfolded the protein structure, stretching and disrupting disulfide bonds (S-S) within the protein molecules, resulting in enhanced SH content (16). Our results agree with those of Zhang et al. (46), who reported that the SH content of canola protein isolates increased after PEF treatment compared with untreated samples. Additionally, in the combined treatment, the MR mechanical force disrupted protein aggregates and promoted protein unfolding, while the PEF treatment partially weakened and disrupted disulfide bonds, thereby further unfolding the protein structure. In addition, these combined treatment effects exposed previously buried SH groups and enhanced the accessibility of reactive sulfhydryl residues. Further, the dissociation of aggregates and cleavage of some disulfide linkages may have contributed to the formation of more free SH groups. Finally, the combined effect of MR and PEF increased the SH content by promoting structural unfolding and reducing protein aggregation.

Furthermore, *H*_0_ values of the treated RPSPI samples are shown in Figure 5B. The findings revealed that the *H*_0_ values of the treated RPSPI samples increased to 498.3 and 590.4 following MR and PEF treatments, respectively, compared with 410.6 for the control sample. In comparison, the combined treatment MR-PEF15 showed the highest *H*_0_ value (650.6) among all treated samples. The modifications in *H*_0_ values indicated that MR-PEF treatment altered the exposure of hydrophobic regions on the protein surface, which may reflect partial unfolding of conformational rearrangement of RPSPI. These findings should be considered together with the intrinsic fluorescence results. Although the fluorescence emission maximum remained within the 335–400 nm after treatment, the fluorescence intensity reduced relative to the control samples, indicating fluorescence quenching and changes in the microenvironment or accessibility of tryptophan residues. On the other hand, rather than suggesting that the tertiary structure stayed entirely unchanged, the lack of a noticeable shift in the maximum emission indicates that the polarity of the surrounding environment of the fluorophores was not significantly affected. The increase in *H*_0_ values and fluorescence can be explained as complementary evidence of treatment induced conformational rearrangement, involving changes in surface hydrophobic group exposure and surrounding environment of intrinsic fluorophores. The results suggest that MR-PEF induced partial structural modification of RPSPI rather than complete disruption of its tertiary structure. *H*_0_ is a sensitive indicator of tertiary structural alterations in protein and shows the number of amino acids exposed to the surface (47). The increase in *H*_0_ value after MR treatment might be linked to a significant reduction in particle size, enhancing solvent accessibility, decreasing aggregation, and eventually exposing more hydrophobic domains at the surface. Similarly, a study reported that high-speed homogenization of soybean protein isolates resulted in higher *H*_0_ values for the treated samples than for the untreated samples (12).

On the other hand, an increased *H*_0_ value after PEF treatment may be due to disruption of hydrophobic interactions within protein molecules and particles, leading to protein unfolding and partial interaction of hydrophobic cavities (48). Our results agree with Li et al. (49) and Wang et al. (16), who reported that the *H*_0_ value of soybean and soy protein isolates increased after PEF treatment compared to the control samples. Additionally, combined treatments were more effective at enhancing *H*_0_ values; this could be attributed to the mechanical force of MR and the electrical energy of PEF, which resulted in higher *H*_0_ values in the treated samples. Interestingly, the combined treatment of MR-PEF20 samples showed reduced in *H*_0_ value as compared to MR-PEF15, when the PEF treatment increased to 20 kV/cm, which showed that higher intensity of PEF might induce aggregation through hydrophobic interaction or disulfide bonds, exposing the hydrophobic groups on the protein molecules (50).

### 3.11 Emulsifying activity index (EAI) and emulsifying stability index (ESI)

Figure 6A represents the impact of MR, PEF, and their combined treatment on EAI and ESI content of treated RSPS samples. The results showed that the treatments significantly enhanced both EAI and ESI values compared with control samples, with the greatest increase in the combined treatment MR-PEF15. Studies revealed that results in surface hydrophobicity and solubility are directly or indirectly linked to better emulsification properties of protein (51). In addition, the increased EAI and ESI values after MR treatment could be attributed to reduced particle size, partial tertiary structure unfolding, and enhanced *H*_0_ value and surface charge. These structural changes in the protein promote discussion to a freshly formed water-oil interface and maintain interfacial coating, resulting in evenly coated tiny droplets and improved EAI and ESI (40). Our results agree with Thakur et al. (11), who reported that the EAI and ESI values of MR-treated sorghum protein isolates were higher than those of untreated samples. In the case of PEF, the treatment increases the EAI and ESI content, as the treatment affects the protein outer surface, which is highly hydrophobic, enhancing protein-water interactions and decreasing tension and adsorption force at the oil-water interface (24, 52). Our results agree with those of Zhang et al. (45), who reported that the EAI and ESI content of the canola protein increased after PEF treatment compared to untreated samples. Additionally, the combined treatment of MR and PEF effectively enhanced the EAI and ESI values; mechanistically, the mechanical force and cavitation of MR, and the electrostatic force of PEF, combined to partially unfold protein molecules. These structural changes exposed both hydrophobic and hydrophilic groups, allowing the protein to adsorb more quickly and efficiently at the oil-water interface. At the same time, enhanced surface activity increased emulsion formation, which caused higher EAI, whereas the formation of a higher flexible and cohesive interfacial protein layer enhanced resistance against droplets, resulting in higher emulsifying stability ESI. On the other hand, both EAI and ESI values were slightly reduced when the PEF treatment voltage increased to 20 kV/cm compared to MR-PEF15 but remained higher than those of other treatments. This could be attributed to the higher PEF intensity, which increased protein isolate aggregates and reduced the solubility and stability of oil-water interfacial layers, resulting in a minor reduction in both EAI and ESI values (22, 24). A similar trend was observed in the study by Wang et al. (16), who reported that the combination of pH shifting and PEF treatment at 10 kV/cm improved the functional properties of the soy protein isolates, whereas enhancing the PEF treatment to 20 kV/cm resulted in a slight reduction in functional properties.

**Figure 6:**
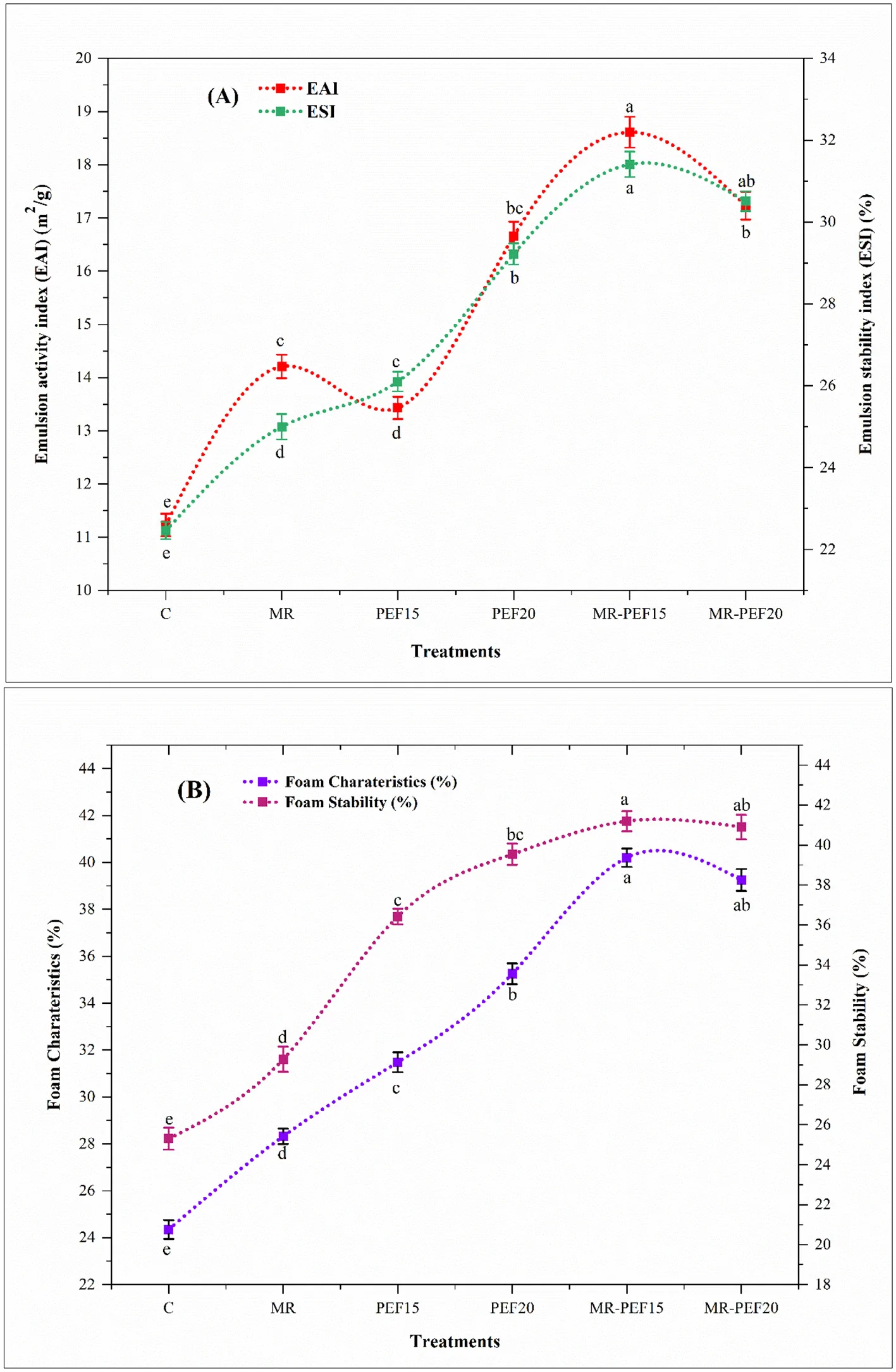
EAI and ESI **(A)**, and FC and FS **(B)** of RPSPI after MR, PEF and their combined treatmen; C, Control; MR, Micronization; PEF15, 15 kV/cm; PEF20, 20 kV/cm; MR-PEF15, MR+15kV/cm; MR-PEF20, MR+20 kV/cm, while the different letters (a-e) above the bars show the significant difference between treatments and control (Tukey's HSD, *p* < 0.05).

### 3.12 Foam capacity (FC) and foam stability (FS)

Foam capacity (FC) represents the ability of proteins to rapidly adsorb and spread at the air-water interface, facilitating the formation of foam, whereas foam stability (FS) reflects the ability of proteins to form a cohesive and viscoelastic interfacial film that maintains the structure and volume of the formed foam over the defined period (53). Figure 6B shows the effect of MR, PEF, and their combined treatment on FC and FS values of RPSPI. The results show that all treatments significantly increased FC and FS values after treatment compared with control samples. However, significant and effective improvements in FC and FS values were observed in the combined treatment MR-PEF15. The increase in FC and FS values after MR treatment could be attributed to reduced particle size, breaking of loose aggregates, and enhanced surface-active protein residues that effectively adsorb to the newly formed air-water interface during whipping (54). Our findings agree with those of Thakur et al. (11), who reported earlier that micronization significantly enhanced the foaming properties of sorghum and RPSPI. On the other hand, the increase in FC and FS values after PEF treatment could be attributed to the concentration of SH groups, which helps the formation of foam films (55). Our study outcomes agree with those of quinoa protein isolates (24) and soy protein isolates (16), revealing that PEF treatment significantly enhanced the foaming properties of treated samples compared to untreated samples.

Additionally, significant improvement in foaming properties after combined treatment could be attributed to MR-PEF, which enhanced adsorption and intermolecular interactions, promoting the formation of a strong cohesive viscoelastic film around the air bubbles, resulting in higher FC and FS values. In contrast, when the PEF voltage increased to 20 kV/cm, the FC and FS values were slightly reduced but remained higher than those of the other samples treated. The minor reduction in FC and FS values could be attributed to irregular forces generated during PEF processing, which reverse polarity and increase protein-protein interactions, thereby decreasing protein stability, leading to less protein absorbed at the air-water interface and eventually, decreased foaming properties (56).

## 4 Conclusion

This study concludes that MR, PEF, and their combined treatment enhance the functional and structural properties of RPSPI. The finding demonstrated that combined treatment of MR and PEF significantly altered the tertiary structural characteristics of RPSPI. Mechanistically, both electrical and mechanical forces significantly altered PS distribution and induced heat shear, loosening the tertiary structure and partially unfolding it, exposing the hydrophobic area and -SH groups. Additionally, its enhanced foaming and emulsifying properties, accelerated adsorption at interfaces, and generated new groups by increasing surface area and changing the negative surface charges. FTIR analysis with Amide I deconvolution revealed that MR and PEF treatments induced significant secondary structural rearrangement of RPSPI proteins. These conformational changes, together with reduced particle size and PDI values, improved protein dispersion and functional properties, with the combined MR-PEF treatment showing the most pronounced structural modification. The X-ray diffraction analysis revealed changes in crystalline structure and crystallinity, while DSC results demonstrated increased denaturation temperatures after treatment, indicating enhanced thermal stability of RPSPI. While the SEM micrographs showed significant structural modification and fragmentation. These findings confirm that MR and PEF are green technologies for value-added utilization of plant protein by enhancing the techno-functional properties of protein isolates and turning them into valuable products. Finally, future research should focus on optimizing treatment conditions, the scalability of the treatment for industrial-scale applications, and the application of the RPSPI in real food systems.

## Data Availability

The raw data supporting the conclusions of this article will be made available by the authors, without undue reservation.
